# A comparison of the performance of normal middle social class Egyptian infants and toddlers with the reference norms of the Bayley Scales -third edition (Bayley III): A pilot study

**DOI:** 10.1371/journal.pone.0260138

**Published:** 2021-12-02

**Authors:** Ebtissam M. Salah El-Din, Zeinab M. Monir, Manal A. Shehata, Marwa W. Abouelnaga, Mones M. Abushady, Mai M. Youssef, Hala S. Megahed, Samar M. E. Salem, Ammal M. Metwally

**Affiliations:** 1 Child Health Department, Medical Research and Clinical Studies Institute, National Research Centre, Giza, Egypt; 2 Department of Community Medicine Research, Medical Research and Clinical Studies Institute, National Research Centre, Giza, Egypt; Aga Khan University, PAKISTAN

## Abstract

**Background:**

Developmental assessment is an important facility for early detection and intervention of developmental delay in children. Objective: to assess the performance of a sample of middle social class Egyptian infants and toddlers on Bayley Scales of Infant and Toddler Development-third edition (Bayley III), and to compare their cognitive, motor, and communication scores with that of the reference norms.

**Methods:**

It was a cross-sectional pilot study, included 270 children aged 18–42 months. Mothers filled a questionnaire including questions about family socioeconomic background, perinatal history, and family history. Physical examination and growth assessment of children were performed. Developmental assessment of cognitive, language and motor skills was performed using the Bayley III scales and compared the American norm scores with the Egyptian mean scores.

**Results:**

The mean cognitive, language and motor composite scores were 92.5+18.5, 91.76+ 15.6, and 95.67+18.9 respectively. All were lower than the American mean (100+ 15) with highly significant differences. About one-fourth of the enrolled Egyptian children had below-average composite scores according to the US cutoff point. The ranks of Egyptian children on the American versus the Egyptian percentile curves were significantly different.

**Conclusion:**

Mean values of all assessed developmental domains of Egyptian children are within the norm-referenced average of Bayley III, but lower than the recorded American mean. Assessing Egyptian children according to the American norms may result in overestimating developmental delay. This pilot study raised the question about using different cutoff points suitable for the developmental trajectory of Egyptian children. Answering this question needs further studies on Bayley-III after cultural adaptation and standardization, using a larger, more diverse, and representative sample of the Egyptian population.

## Introduction

Early childhood is a great opportunity for optimal brain development, but it is also a time of vulnerability. In these first years, development in language, cognition, motor, and socio-emotional domains occurs quickly and collaboratively [1].

Dysregulation of neural systems can result in disruption of normal development, which may lead to pronounced neurocognitive deficits, delays in language, or social-emotional functioning, poor academic achievement, and poor productivity in adulthood [2].

The prevalence of disability in Egypt has varied widely, due to the lack of regular and constant developmental assessment [3, 4]. None of this reported prevalence seems accurate, in addition, there is a lack of data on children below 6 years.

Early detection of children with developmental disabilities, along with early childhood intervention, increases the opportunities for children to enhance their developmental capacity and functioning and ultimately their quality of life and social involvement [5, 6].

Identification of infants who need early intervention requires the use of a valid developmental diagnostic assessment tool. No Egyptian tool has been established yet. The Bayley Scales of infant development; a tool designed and norm-referenced in the USA; is a commonly used psychometric tool for assessing the development of children between 1 to 42 months of age. It has proven to be a reliable diagnostic method and is the gold standard in infant evaluation for recognizing children with early developmental delays [7–9]. The Bayley scales have been used frequently in various countries such as the Netherlands [10], Iran [11], Australia [12], and Asian countries [9].

However, cross-culture difference has been reported [13], in different countries [14, 15]. It is important to acknowledge that an instrument that works well for one population may not work as well for another population, because different populations may have different rates of development [16].

A technical consultation was carried out in 2015, to develop a National Disability, Health, and Rehabilitation Plan (NDHRP) for Egypt. The consultants recommended developing feasible and culturally accepted tools in health reporting and clinical assessment of disability and functioning by a team of experts [17].

WHO recommends that, in low- and middle-income countries with a high prevalence of conditions that are hazardous to child health and development (such as malnutrition, low birth weight, chronic infections, parasitic infestations, iron-deficiency anemia, and perinatal complications), references for monitoring growth and development should be based on a sample of healthy children without these risks, rather than on a geographical population [16]. In Egypt, a significant proportion of the population, almost 30 percent, are considered middle class. In contrast to the poor and at-risk population, the middle social class has higher education, more resources, and better integration to essential services and pays a large portion of income on education and health [18].

Accordingly, the objective of this study was to assess the performance of a sample of normal middle social class Egyptian infants and toddlers in the age range of 18 months to 42 months on the cognitive, motor, and communication scales of Bayley-III, comparing the Egyptian scores with the US norm scores. Our optimum goal is to create our own Egyptian norms of child development for proper assessment and precise intervention.

## Materials and methods

Study design: It was a cross-sectional pilot study, included 270 children aged 18–42 months divided into 4 age groups with six months intervals.

### Sample size

A sample size of 57 participants (rounded to 60) for each age category produces a two-sided 95% confidence interval with a distance from the mean to the limits that are equal to 3.980 when the estimated standard deviation is 15.000. A total of 264 to be rounded to 270 (240 with added 10% expected losses) will ensure the adequacy of the sample size [19].

### Selection of participants

Egyptian children included in this study had the following criteria: gestational age of at least 37 weeks, birth weight of at least 2500 grams, no physical or mental health issues, of normal weight and height for their age, and middle social background. The exclusion criteria were prematurity or low birth weight, genetic, congenital, or metabolic disorder, history of perinatal complications as intracranial hemorrhage, of chronic disease, of severe sensory impairment (auditory or visual) and if the child is severely malnourished (If height-per age z-score (HAZ), or weight-per age z-score (WAZ) or body mass index per age z-score (BAZ) is less than -2).

Two hundred and seventy infants and toddlers of both sexes were included in this study, over a period of one year, they were divided into 4 age groups with six months intervals (18–24 months, 25–30 months, 31–36 months, and 37–42 months) with a minimum of 65 participants in each.

The study was conducted at the Developmental and Behavioral Assessment Clinic at the Center of Excellence, National Research Center (NRC). Infants and children were recruited from nearby nurseries and the Centre of Excellence Pediatrics Clinics.

### Ethical issues

The study proposal was approved by the Medical Research Ethical Committee of the National Research Centre. Mothers or caregivers were informed about the purpose of the study and their permission in the form of written consent was obtained. Mothers and children were identified by a serial number and the information at the individual level was kept strictly confidential.

### Procedures

- **Background questionnaire:** Mothers/caregivers answered a background questionnaire containing questions about family sociodemographic data and child characteristics. Questions about family composition, residence, parental education and occupation, monthly income (for assessment of socioeconomic status according to El- Shakhas [20]).
- **Physical examination and growth assessment:** Infants and toddlers were thoroughly examined by expert pediatricians. Growth assessment was performed using anthropometric measurements including weight (Kg), height (cm), and head circumferences (cm), following the recommendation of the International Biological Program [21]. Weight for age Z score (WAZ), Height for age Z score (HAZ), and BMI- Z-scores were calculated based on the WHO growth standards [22] with the help of the Anthro-plus Program of PC.
- **Assessment of development:** Bayley Scales of Infant and Toddler Development (Bayley III), developed by Nancy Bayley in 2006, was utilized to assess the development of infants and toddlers between the age scopes of 1 month to 42 months [23]. Bayley-III covers five developmental domains. Cognitive, motor, and language domains are administered with the child’s interaction, while social-emotional and adaptive behavior domains are administered with parent questionnaires. In this study, social-emotional and adaptive behavior domains were not assessed.
- The Cognitive Scale included items that assess sensorimotor development, exploration and manipulation, object relatedness, concept formation, memory, and other aspects of cognitive processing.
- The Language Scale is composed of receptive communication and expressive communication items. The Receptive Communication subtest included items that assess preverbal behaviors; vocabulary development and items that measure children’s social referencing and verbal comprehension. The Expressive Communication subtest included items that assess preverbal communication, such as babbling, gesturing, joint referencing, and turn-taking; vocabulary development, such as naming objects, pictures, and attributes.
- The Motor Scale is divided into the Fine Motor subtest and the Gross Motor subtest. Fine motor skills included items that measure skills related to visual tracking, reaching, object manipulation, and grasping. The Gross Motor subtest assessed static positioning (e.g., sitting, standing); dynamic movement (e.g., locomotion, coordination); balance; and motor planning.

Scoring for every item is either 1 (credit) or 0 (no credit). Scores available include raw scores, scaled scores, composite scores, percentile ranks, and confidence intervals.

The measure with a series of developmental play tasks took between 45–60 minutes to administer. Raw scores of successfully completed items were converted to scaled scores and composite scores. The scores obtained by toddlers were used to determine their performance compared with norms taken from typically developing children. The composite scores are scaled to a metric with a mean of 100 and a SD of 15, and a range from 40–160. The norm-referenced average is from 85–115.

The instruction manual was translated into Arabic, the native language, by the researchers, and back-translation to English was done by an independent professional person. Both English versions were comparable.

The Bayley III assessment was done by well-trained pediatricians who were experienced in using the Bayley-III and in early child development. Inter-rater reliability between examiners was estimated, and the average kappa was 0.80.

### Statistical analysis

Data analysis was performed using Statistical Package for the Social Science (SPSS) version 21 (SSPS Inc, Pennsylvania, USA). Continuous data were expressed as mean ±SD and compared using t-test, while categorical data were expressed as frequencies and percentages using Chi-square analysis. ANOVA test was used to analyze the statistical difference between mean scores of the three subtests. P-value was considered statistically significant at p<0.05. The mean (standard deviation) of the original normative Bayley population was 100± 15, and the original normative population data had a bell-shaped distribution. An Egyptian percentile curve was created depending on the participants’ scores.

## Results

A total number of 270 infants and toddlers were recruited. The mean (SD) age at enrollment was 28.01+ 9.162 months, and about 43.7% were female. All the included families belonged to the middle social class. About 50% of mothers were University educated. Most of the mothers (69.7%) were housewives. Nearly 41% of the participants were admitted to the nursery in the first year of life. The mean weight, height, and head circumference were all within normal range and the participants had normal BMI for their age and sex.

**Mean and SD of Bayley III** cognitive, language, and motor composite scores and percentile rank were shown in (Tables 1 and 2). The mean cognitive, language, and motor composite scores were 92.5±18.5, 91.76± 15.6, and 95.67±18.9 respectively. All these values are within the norm-referenced average of Bayley III (85–115). However, all were lower than the American mean (100± 15) and the differences were highly statistically significant.

**Table 1:** The difference between mean Egyptian and mean American composite scores in each subtest of Bayley Scales.

Composite score	Test Value = 100*					
	Mean Egyptian	Std. Deviation	Sig. (2-tailed)	Mean Difference	95% Confidence Interval of the Difference	
					Lower	Upper
Cognitive composite score	92.5431	18.44678	<0.001	-7.45690-	-9.8431-	-5.0707-
Language total composite score	91.7566	15.59995	<0.001	-8.24336-	-10.2882-	-6.1985-
Motor total composite score	95.6652	18.88092	0.001	-4.33480-	-6.8042-	-1.8654-

*The mean American composite score of each subtest is 100.

**Table 2:** Comparison between Bayley percentile rank and percentile of the Egyptian studied sample.

	Group	Mean	SD	t-test	p
Cognitive percentile rank	Bayley percentile rank	35.6281	27.30338	-4.173	<0.001*
	Egyptian percentile rank	46.6388	29.42271		
Language percentile rank	Bayley percentile rank	34.4500	25.24403	-5.612	<0.001*
	Egyptian percentile rank	48.6562	28.48024		
Motor percentile rank	Bayley percentile rank	41.1384	31.29743	-2.665	0.008*
	Egyptian percentile rank	48.6562	28.48024		

*p < 0.05.

The mean language composite score was the most affected by 8.2 points less than the American mean, while the mean motor composite score was the closest to the American mean by a difference equals to 4.3points (Table 1).

**Fig 1:**
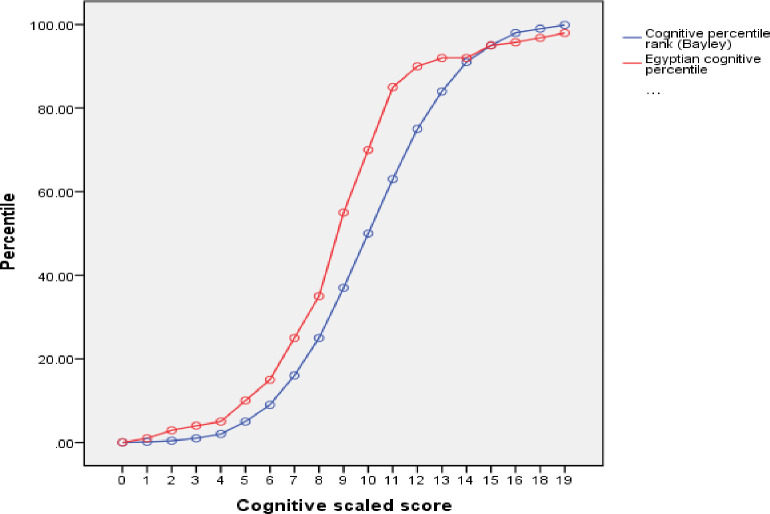
Egyptian versus American percentiles of the cognitive domain.

**Fig 2:**
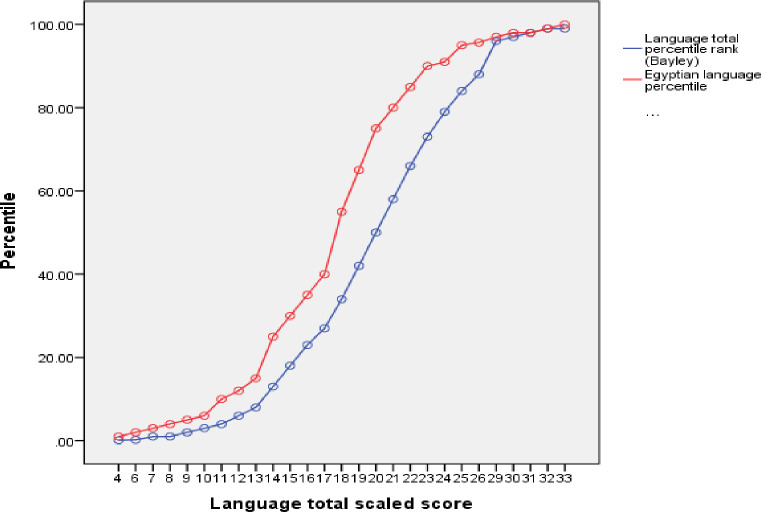
Egyptian versus American percentiles of language domain.

**Fig 3:**
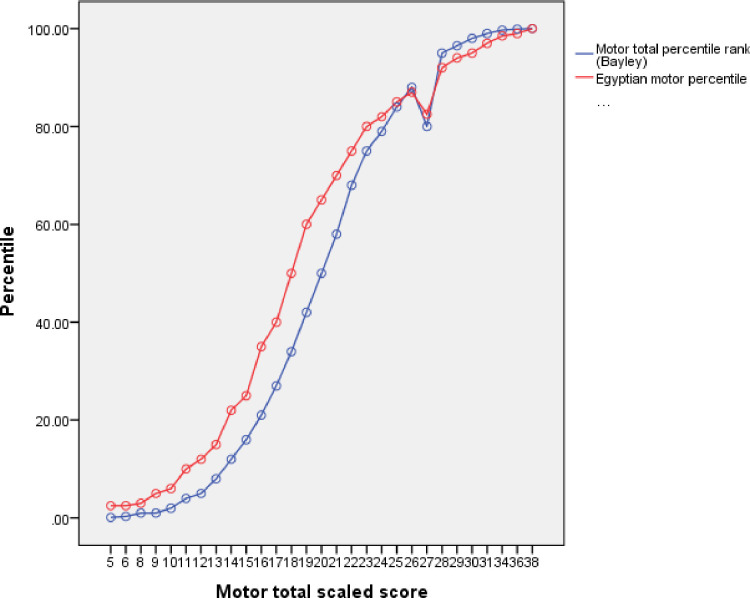
Egyptian versus American percentiles of the motor domain.

Similar significant differences were observed between American and Egyptian percentile rank (Table 2). The differences between the ranks of Egyptian children on the American and the Egyptian percentile curves were shown in (Figs 1–3). As an example, the mean cognitive percentile rank was 35.6^th^ on the American percentile curve, while this rank was 46.6^th^ on the created Egyptian percentile curve.

**Fig 4:**
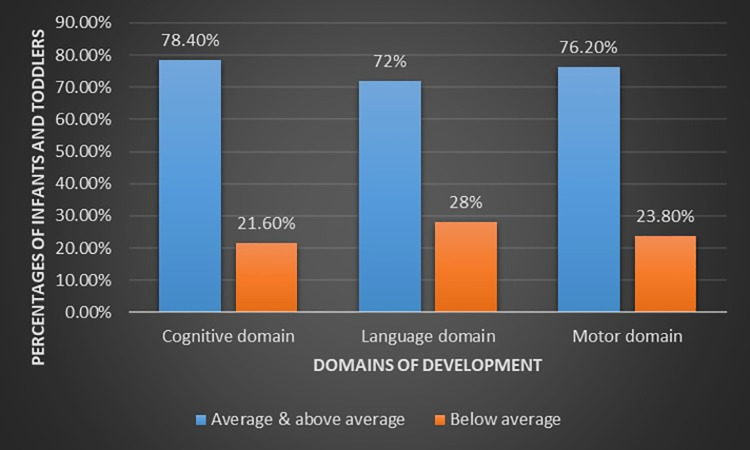
Classification of participants into two groups (Average & above average group and below average group) according to the cutoff point of composite score of Bayley Scales.

Participants of the present study were categorized into two groups according to their performance on the Bayley III scales (Fig 4). The first group has got an average or above-average composite score equal to or above 85. The second group has got a below-average composite score (less than 85). Clinically this latter group is considered at risk of developmental delay. The percentage of the below-average population in this study was 21.6%, 28%, and 23.8% in cognitive, language, and motor domains, respectively.

## Discussion

The Bayley scales have been used for assessing the development of infants and young children in several countries such as the Netherlands [10], Australia [12], and Asian countries [9].

Strong reliability and validity of the scale have been reported in the literature [13, 24, 25]. Though, cross-culture variance has been stated in diverse populations [10, 15, 24].

The focus of this pilot study is to assess the performance of a sample of middle-class Egyptian children on cognitive, language, and motor developmental scales of Bayley III and compare the resultant Egyptian scores with the US norm scores.

Our results demonstrated that means of all the studied subtest values were significantly lower than the American mean (100+ 15), although they were within the norm-referenced average of Bayley III (85–115). The mean language composite score was mostly affected by 8.2 points less than the American mean, while the mean motor composite score was the nearest to the American mean by a difference equals to 4.3points.

In accordance, previous studies from various countries all over the world, have reported differences when comparing the performance of Bayley III with the American norms. A Dutch study reported significant variations between the performance of Dutch children compared to the American standards, they have demonstrated that using the US norms caused an over-referral as regards the gross motor development, and under-referral as regards other domains skills [10].

Godamunne and his colleagues [9] evaluated the performance of Sri Lankan children on cognitive and motor subtests of Bayley scale in comparison to the American norms at 12 months, their results showed significantly higher cognitive skills and lower gross motor skills, and at 24 months, there were significantly lower cognitive scores. Also, children from rural Nepal had lower scores regarding the cognition and motor domains in comparison to the American norms [25]. Nevertheless, another Nepalese study reported that the performance of Nepalese children in all domains was comparable to the American norms, but was significantly lower regarding the language subtests, cultural modulations, and tool standardization was recommended by the investigators to establish the validity and reliability of the test [26]. In the same context, a Danish study found that the Cognitive and Motor Scores from the Danish children showed insignificant differences from US norms, whereas, the Language scores were significantly higher. The authors suggested that these differences may be due to a low-risk study sample and highly educated parents [15].

Similar significant differences were observed between American and Egyptian scaled scores and percentile rank. As an example, the mean cognitive percentile rank was 35.6th on the American percentile curve, while this rank was 46.6th on the created Egyptian percentile curve. These findings raise the doubts about the appropriateness of Bayley III as a tool for developmental assessment in Egyptian infants and children. As the healthy typically developing children were falling within the normal range so this tool could easily be used to assess for developmental delays. Also, the way forward should include validation and reliability testing of the tool for Egyptian children of all socioeconomic groups.

Various factors could explain the difference between the mean scaled and composite values in the current study from the American norms. The United States is a developed country, so its population could have an advantage in comparison to that of Egypt, a developing country. Since the two populations vary culturally, geographically, ethnically, and socio-economically, their performance on assessments can be anticipated to vary, as these factors are well-known to impact development. Culturally informed literature suggested that psychomotor development in infancy is not determined solely by biological factors but rather is affected systematically by childrearing practices, which differ by culture. When African infants are reared following European American practices, they do not show the motor advances of peers reared using traditional African practices. Even domains of infantile development under apparent biological control are plastic, within parameters, to parenting and culture [27]. Thus, the low score of children in all developmental domains could also be due to child-rearing practices in Egypt, as well as other social and environmental factors [28]. In a study about parenting in the upper and middle class in Egypt, Egyptian parents showed insufficient knowledge about their children’s development and demonstrated discrepancies in parenting and discipline styles. Mothers also had a lot of provocations with managing their time and maintaining a balance between their work and their children and homes [29].

We believe that lack of a stimulatory environment, besides harsh and restrictive parenting practices may have a harmful effect on the development of Egyptian children. In accordance, recent Egyptian studies proved that all forms of child maltreatment, even mild forms, are associated with increased levels of internalizing and externalizing problems in offspring. In addition, child neglect was linked to social, thought, and attention problems as well as externalizing and internalizing disorders [30, 31].

In addition, a recent Egyptian intervention study determined the effect of maternal training programs on infant development. In this program, mothers learned how to promote interaction with their children and to stimulate their development. The study revealed that maternal social support is one of the most important predictors for normal development [32].

Moreover, regional variations in brain development may also explicate differences in performance of all domains, as there are different rates of development across populations [33].

From another perspective, some risk factors, which are known to be closely related to developing countries, were found to be involved in brain development. For example, nutritional deficiencies, that were identified as detrimental risk factors for development, including macronutrients (e.g., protein, long-chain polyunsaturated fatty acids, and glucose) and micronutrients (e.g., iron, iodine, zinc, and vitamins), their effect on the brain may extend from conception to about 3 years of life, the basis for the duration brain functions are established [34, 35]. Other individualized factors such as low birth weight, older paternal age, and lower maternal education, were identified as risk factors affecting deviant development [36, 37]. Although researchers of this study tried to avoid most of these risk factors, the possibility of micronutrient deficiencies with their detrimental effects cannot be excluded.

Participants of the present study were categorized into two groups according to their performance on the Bayley III scales. Nearly 25% of the study population had a below-average composite score. On the other hand, in the Bayley III normative population which included 10% clinical cases, the percentage of the population who had scores below 85 was 15.86% [38]. This raises a question about the most appropriate cut-off for defining below average or at risk of developmental delay in the Egyptian population. The use of the Western norms and cutoff point of Bayley III will result in over-referral, as more children will be identified with a developmental delay in comparison to the USA- norms. Additionally, the cross-cultural bias presented when assessing development in populations with considerably different child-rearing practices can be addressed by the establishment of local norms [24].

We have some limitations in this study: the sample size was small owing to the longtime of assessment, restricted to the middle social class of Cairo residents, and was neither representative to all governorates nor to other social classes of Egypt. Investigation for micronutrient deficiencies was not accessible. So, results cannot be generalized. Still, this pilot study is paving the way for the following large and representative studies. The strength of this study was the precise inclusion and exclusion criteria. This allowed the recruitment of healthy middle-class individuals who were expected to have normal development. The exclusion of lower social class subjects escaped the supposed impact of malnutrition and poverty on development.

**In conclusion,** this study reveals the importance of population-specific norms for the interpretations of developmental assessment results. Our findings had demonstrated significant differences between Egyptian scores in comparison to US norms of Bayley-III. Thus, using the American norms may result in over-referral. More Egyptian children may be diagnosed with developmental delay. This pilot study raised the question about using different cutoff point suitable for the developmental trajectory of Egyptian children. Answering this question needs further studies on Bayley-III after cultural adaptation and standardization, using a larger, more diverse, and representative sample of the Egyptian population.

## Supporting information

S1 File Raw data of the study. (XLSX)
